# Building a Pediatric Patient Registry to Study Health Outcomes Among Transgender and Gender Expansive Youth at a Rural Gender Clinic

**DOI:** 10.1089/trgh.2018.0023

**Published:** 2018-12-18

**Authors:** Jane O'Bryan, Kimberly Leon, Carolyn Wolf-Gould, Melissa Scribani, Nancy Tallman, Anne Gadomski

**Affiliations:** ^1^ Bassett Research Institute, Bassett Medical Center, Cooperstown, New York.; ^2^ Columbia-Bassett Program, Columbia University Vagelos College of Physicians and Surgeons, Cooperstown, New York.; ^3^ The Gender Wellness Center/Susquehanna Family Practice, A.O. Fox Hospital, Bassett Healthcare Network, Oneonta, New York.

**Keywords:** gender, pediatric, registry, transgender

## Abstract

**Purpose:** Significant knowledge gaps regarding outcomes of gender-affirming therapy in transgender (TG) and gender expansive (GE) youth impede an evidence-based approach to these patients. The Gender Wellness Center (GWC) Pediatric Patient Registry was established in 2017 to enable systematic, longitudinal research to describe the physical, mental, and quality-of-life outcomes of these youth.

**Methods:** All TG/GE youth, ages 8–21 years, presenting to the GWC were recruited on site. Ten research questions guided the creation of data fields. The following 131 variables were abstracted from electronic medical records: demographics, weight, height, body mass index, gender identity, sexual orientation, coexisting diagnoses, substance use, Tanner stage, sexual activity, medications, fertility preservation, Gonadotropin Releasing Hormone (GnRH) analog use, hormone therapy, surgery, and related outcomes. Health-related quality of life is assessed using the Child Health Questionnaire-87 for ages <18 and the Short Form-36 for ages 18–21.

**Results:** To date, 139 TG and GE youth (90% white and 93% non-Hispanic), have enrolled in the registry. Average age at enrollment was 17.5 years (±3.1, range: 8–21). Two-thirds of youth identified on the trans masculine spectrum (*n*=90), 28.8% identified on the trans feminine spectrum (*n*=40), and 6.5% identified as nonbinary/gender nonconforming (*n*=9). Nearly, all youth had socially transitioned (*n*=121, 87.7%) and were medically transitioning (*n*=123, 89.1%).

**Conclusion:** As one of the first rural-based registries, the GWC Registry has helped to delineate health outcomes attributable to gender-affirming care in a unique patient population of TG/GE youth. Our results will be used to describe treatment outcomes that will contribute to evidence-based guidelines.

## Introduction

An increasing number of gender expansive (GE) youth are seeking medical services for the treatment of gender dysphoria,^1–3^ the distress arising from the incongruity of assigned sex at birth and gender identity.^4,5^ Gender dysphoria may manifest as mental health problems, including anxiety, depression, low self-esteem, high-risk behaviors, and suicidality.^6–14^ Significant knowledge gaps exist in nearly all aspects of the clinical management of gender dysphoria in youth,^2,15^ contributing to uncertainty and practice variability among clinicians caring for GE youth.

Existing recommendations for the treatment of gender dysphoric youth and medical transition are based on a limited number of longitudinal studies with relatively small sample sizes.^16–18^ The urgent and unmet need for scientifically rigorous, longitudinal studies of both GE youth and adults has been consistently emphasized in the transgender (TG) health literature.^2,19,20^

### Registries as a research tool in pediatric and TG health

Registries are one of the most effective research tools for studying rare diseases, and underrepresented or vulnerable populations. Patient registries allow clinicians and researchers to collect systematic information about specific patients or diseases, increasing understanding of conditions and treatment outcomes.^21^ Registries support quality measurement, provide feedback to clinicians and institutions for quality improvement, facilitate clinical research, and enable evaluation of healthcare access and disparities. Creation of a national TG registry and conducting large multicenter cohort studies have been identified as research priorities in the field of TG health.^19,22^

### The Gender Wellness Center Pediatric Patient Registry

The Gender Wellness Center (GWC) of the Bassett Healthcare Network is a nationally recognized, rural-based, multidisciplinary center that offers gender-affirming medical, mental health, and surgical care to TG and GE children, youth and adults in Upstate New York. The GWC is embedded within Susquehanna Family Practice, located in Oneonta, New York, and has been providing gender-affirming primary care and hormone therapy for adults since 2007, and for youth, since 2012. The GWC Pediatric Patient Registry, established in 2017, has three purposes: (1) to enable systematic, in-depth study (descriptive, retrospective, and prospective longitudinal) of the pediatric patient population served by the GWC; (2) to enhance understanding of the health care needs of TG/GE youth (including physical, mental, and social health and well-being); and (3) to contribute to and fill gaps in the existing evidence based on best clinical practices in gender care.

## Methods

### Standard protocol approvals, registrations, and patient consents

Study procedures were approved by the Mary Imogene Bassett Hospital Institutional Review Board. All subjects or legally authorized representatives (LAR) provided informed consent for study participation. A parent or legal guardian provided informed consent for all patients <18 years of age; patients are reconsented upon reaching the age of majority. Verbal assent was also obtained from youth ages 7–17. Youth ages 18–21 provided informed consent as legal adults.

### Subject population and recruitment

All pediatric patients (defined as <22 years of age) receiving gender-affirming care at the GWC are eligible for inclusion in the registry. There are no exclusions on the basis of gender identity. Prospective participants are approached on site at GWC appointments. The recruitment process is initiated by the patient's GWC clinician, who briefly explains the registry and its goals during or after the visit. If the patient (and LAR, if applicable) is interested in learning more about registry involvement, a research coordinator for the registry meets with the patient to explain participation, risks, benefits, and all other details, and provides informed consent documents. If the patient (and LAR, if applicable) consents to participate, the patient is assigned a research number, enrolled in the registry, and completes baseline questionnaires.

### Registry design and development

The registry database was designed through an iterative process in collaboration with GWC clinicians. Registry data fields were created using a three-step process. The first step involved a shadowing period by the research team lasting several weeks. GWC clinicians were observed during clinical encounters with patients. The purpose of shadowing each of the clinicians was to understand how patient care and questions about gender identity and transition were handled by different clinicians. It was important to note if and how certain information was collected during clinic visits and how these data were documented in the electronic medical record (EMR), given that the registry was intended to be a systematic data collection tool that included standardized data fields available for most patients.

The second step of the process involved careful analysis of a selection of patient records from the GWC's previous EMR platform, Horizon Ambulatory Care (HAC, by McKesson), which was replaced with Epic in April 2017. The HAC system included free text fields similar to chart notes as well as active problems, family history, social history, immunization records, allergies, medications, encounters, and International Statistical Classification of Diseases and Related Health Problems (ICD) codes, among other categories. Initial visits to the GWC were recorded in the HAC system in semistructured narrative form. There was not a consistent format across narratives or across clinicians, and the narrative format posed a significant challenge for registry data abstraction. In addition, patient intake forms, test results, mental health assessments, and referral letters were scanned into the patient record. The information contained in the scanned documents was not readily extractable from the HAC EMR, posing an additional challenge for data abstraction.

The registry data fields were selected to answer 10 specific research questions. These questions were identified as research priorities by GWC clinicians due to their relevance to gender-affirming therapy and to clinical controversies in TG healthcare. Several of the questions are well-suited to cross-sectional study (e.g., determine the prevalence of self-harm and eating disorders). Other questions can only be addressed by longitudinal data collection (i.e., effects of gender-affirming hormone therapy). The full list of questions is outlined in Table 1.

**Table 1.** Ten Research Questions Prioritized by Gender Wellness Center Clinicians

(1) Is the age at which gender incongruence is first manifested predictive of its persistence/desistence?
(2) Is gender identity stability over time associated with a greater likelihood of persistent gender incongruence?
(3) Does bone density decrease among patients receiving GnRH analogs? And, does bone density return to normal expected levels after treatment with GAHT?
(4) How does expected height compare to actual height among children who receive GnRH analogs and GAHT?
(5) How do pretreatment hormone levels among transgender individuals compare to hormone level norms (matched for Tanner Stage and sex assigned at birth)? Are baseline testosterone levels higher among transgender men compared to norms?
(6) What is the prevalence of self-harm behaviors in this sample of youth?
(7) What is the prevalence of eating disorders in this sample of youth?
(8) Do psychological outcomes (i.e., depression) improve over the course of treatment?
(9) Are there any short-term or long-term adverse effects associated with the use of pubertal blockers and GAHT? Specifically, are there adverse effects associated with Lupron injections?
(10) How do GnRH analogs and/or GAHT affect BMI?

BMI, body mass index; GAHT, gender-affirming hormone therapy; GnRH, Gonadotropin Releasing Hormone.

The list of data fields to be included in the registry was reviewed both from a clinical and analytic standpoint and revised according to the feedback of researchers, biostatisticians, and GWC clinicians to delineate the independent, dependent, mediating, and possible confounding variables. This third stage of the process required translating broader topics into discrete and definable data points that were consistently captured in the EMR across all clinicians. A chart abstraction protocol was developed, piloted, and revised as needed to maximize abstraction efficiency and data integrity.

### Database functionality and design

**FIG. 1.**
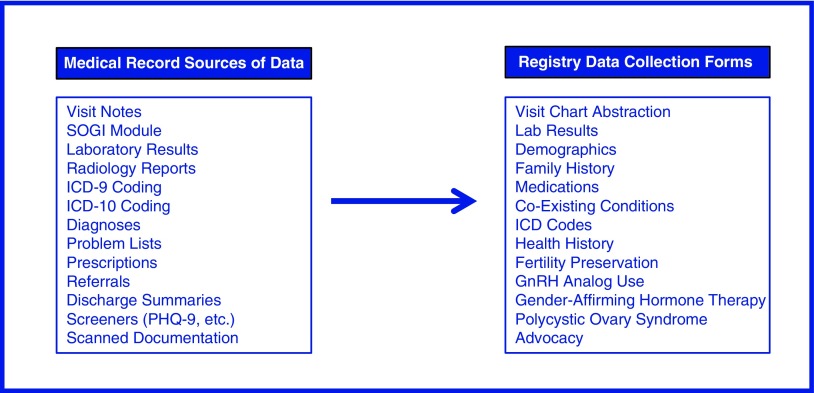
Data collection instruments available for cumulative data capture in the registry database. As information is abstracted from the medical records, it is entered into the appropriate forms. Forms can be completed as many times as necessary throughout longitudinal follow-up.

**FIG. 2.**
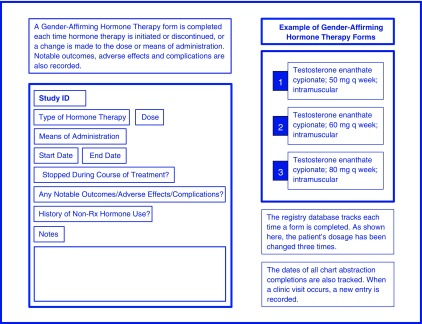
Repeating data collection instruments for longitudinal follow-up. This figure illustrates how repeating data collection instruments can be utilized to track changes over time. The repeating function is useful for data fields such as medication utilization, because medication data change frequently (i.e., new medications added, medications discontinued, and dosage changes).

The GWC Registry database was built in REDCap (Research Electronic Data Capture), a secure, web-based, and Health Insurance Portability and Accountability Act (HIPAA)-compliant platform.^23^ Study data were collected and managed using REDCap electronic data capture tools hosted at Bassett Medical Center. REDCap is an application designed to support data capture for research studies, providing: 1) an intuitive interface for validated data entry; 2) audit trails for tracking data manipulation and export procedures; 3) automated export procedures for seamless data downloads to common statistical packages; and 4) procedures for importing data from external sources.^23^ Fields were created for both the quantitative and qualitative data elements present in the HAC and Epic EMRs. The database was designed with repeating data elements to allow for longitudinal data collection. Data are recorded in the registry by clinic visit. Separate, nonrepeating instruments were designed for fields that are important to capture, but are not available or appropriate to include for all patients (e.g., fields concerning polycystic ovary syndrome) (Figs. 1 and 2).

### Registry data fields

#### Demographic characteristics

The registry captures date of birth, assigned sex at birth, race, ethnicity, and education. When available, this information is abstracted from the EMR. A baseline questionnaire is also administered at the time of enrollment to fill in any gaps in demographic information such as race, ethnicity, and education level.

**FIG. 3.**
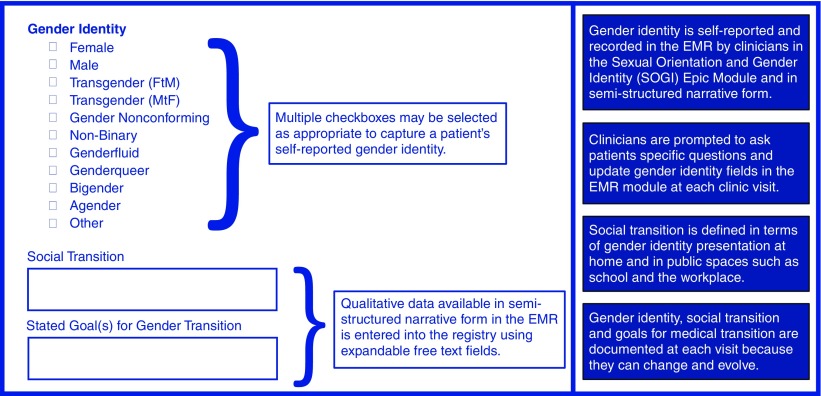
Qualitative and categorical gender identity data collection. This figure illustrates how gender identity information abstracted from the EMR is entered into the appropriate database form. The form is completed after each clinic visit throughout longitudinal follow-up. Qualitative data in the EMR is entered into the registry database using expandable free text fields. EMR, electronic medical record.

Patient information about gender identity and sexual orientation was not recorded in a standardized format in the HAC EMR. These details were usually documented within clinician notes. Although patients were routinely asked to self-identify in terms of sexual orientation or gender identity (SOGI) at clinic visits when the HAC EMR was in use, there was no specific domain for recording SOGI data. After the transition to Epic, a healthcare network-wide SOGI data collection tool was introduced, which prompted clinicians to ask patients four specific questions and update SOGI data by recording patient responses in the EMR module at each clinic visit. SOGI data fields are completed for every visit in Epic records, whereas HAC charts may describe them in semistructured narrative. Figure 3 illustrates how the gender identity aspect of SOGI is captured within the registry database.

As it does change and can evolve, gender identity was captured at each visit, along with social transition status and patient goals for medical transition. Social transition was defined in terms of gender identity presentation (i.e., pronoun use, use of preferred name, and gender presentation, both at home and in public spaces such as at school or in the workplace). Medical transition was defined in terms of gender-affirming therapy, including the use of gonadotropin-releasing hormone analogs (GnRH analogs), hormone therapy, and surgical procedures.

#### Clinical characteristics

Clinical characteristics captured in the registry range from standard elements of health history to fields describing gender identity development and transition, to fields capturing patient advocacy and support. In the broad category of health history, data fields include height, weight, body mass index, Tanner stage, coexisting conditions, ICD-9 and ICD-10 codes, family history, mental health history, sexual health history, drug and alcohol use, and medications. For a patient to qualify as having a mental health problem in the registry, their EMR must have referenced a mental health problem either as an ICD code, a coexisting condition on the problem list or in a note from a mental health specialist.

Registry patients were coded as positive for suicidality if it appeared as an ICD code, coexisting condition or problem, in a note from a mental health specialist or report from a psychiatric institution, or if suicidal ideation, behavior or attempt(s) were recorded in the notes section of the chart by the clinician. Instances of psychiatric hospitalization were most commonly documented in scanned discharge summaries, mental health provider notes, or in referral and progress update letters. History of abuse (including emotional, physical, and sexual) was commonly referenced in EMR notes, particularly when Child Protective Services was involved in case management or intervention.

**FIG. 4.**
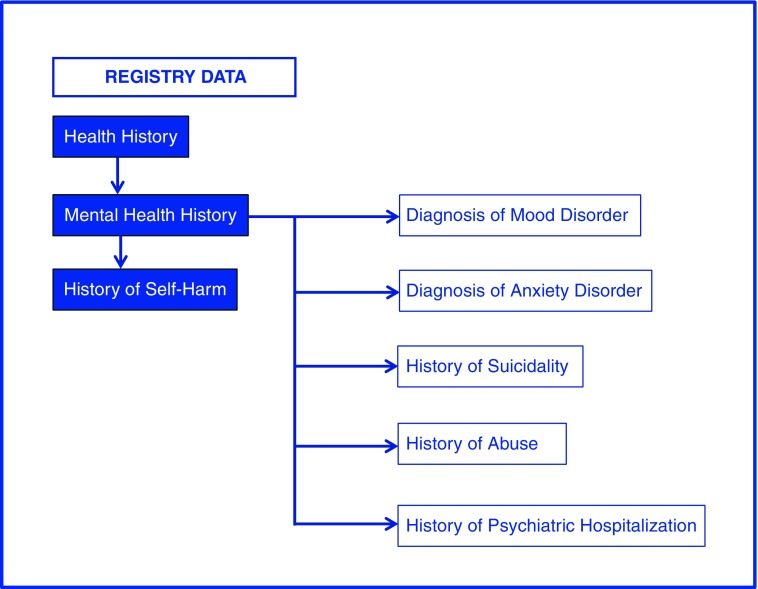
Example of registry data source utilization to answer a research question. This figure illustrates how registry data can be sourced and utilized to answer one of the ten research questions: “What is the prevalence of self-harm behaviors in this sample of youth?” The data field for “History of Self Harm” is within the major database category “Health History,” subcategory “Mental Health History”. Shown also are other mental health data fields relevant to the question being investigated. These fields are potential mediating and confounding variables that should be included in the analysis. The relevant data fields are selected and exported from the REDCap database for analysis.

**FIG. 5.**
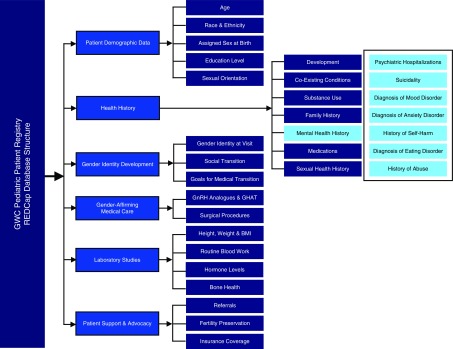
Registry database—major categories and subcategories. This figure depicts the major categories and subcategories of data fields included in the Registry. A total of 131 data fields, including demographic and clinical variables, are abstracted and entered into REDCap. The major categories include: (1) Patient Demographic Data; (2) Health History; (3) Gender Identity Development; (4) Gender Affirming Medical Care; (5) Laboratory Studies; and (6) Patient Support and Advocacy. Shown here is a breakdown of the data fields contained within the category “Health History,” subcategory “Mental Health.” REDCap, research electronic data capture.

Results of laboratory studies, including blood tests, hormone levels, and bone health studies, were abstracted when available. Finally, advocacy and support for the patient, including referrals to outside mental health providers, social services, school counselors, fertility preservation specialists, and medico-legal advisors were abstracted into the registry. Figure 4 illustrates how registry data can be sourced and utilized to answer 1 of the 10 research questions that drove the creation of the registry data fields. Figure 5 demonstrates the level of detail that is captured for one of the subcategories of these broader data fields, mental health history. A total of 131 variables are abstracted into the registry, combining quantitative and qualitative data elements abstracted from both EMRs.

#### Quality of life measurement

Health-related quality-of-life (HRQoL) assessments can provide insight into patients' self-perceived physical and mental health and may identify healthcare needs of populations. Longitudinal administration of HRQoL surveys in TG/GE youth populations can help detect changes in self-rated health throughout transition and increase our understanding of the impact of gender-affirming therapy. For these reasons, we decided to administer HRQoL assessments to all patients enrolled in the registry at baseline and bienially thereafter. HRQoL is assessed using the Child Health Questionnaire-87 (CHQ-87)^24,25^ for youth <18 years of age and the Short Form-36 (SF-36v2) for youth ages 18–21.^26,27^ The reliability and validity of these surveys has been well documented; indeed, the SF-36 is reportedly the most frequently used patient reported outcomes instrument in clinical trials.^28,29^ The CHQ-87 is a child-report form consisting of 87 items and covers both physical health and psychosocial measures. The SF-36 was developed by the RAND Corporation as part of the Medical Outcomes Study and measures physical and mental health across eight domains and a total of 36 questions.^26,27^ Neither scale includes TG-specific questions, but both provide a global assessment of patient-perceived HRQoL that can be compared to standardized US population norms. Licenses were obtained for the use of both surveys.

### Statistical analyses

Descriptive statistics were used to report patient demographic and clinical characteristics. Categorical variables were summarized using frequencies and percentages. All analyses were conducted using SAS v.9.4.

## Results

### Registry enrollment

Of TG/GE youth receiving care at the GWC, 139 have been enrolled in the registry to date (98% recruitment rate). One patient declined to participate, and two patients were interested in participating, but we were unable to obtain informed consent from a LAR.

### Patient demographic and clinical characteristics

Average age at registry enrollment was 17.5 years (±3.1, range: 8–21 years). Average age at clinic presentation was 16.7 years (±3.0). Ninety percent of participants were white, 93% were non-Hispanic, and 46% reported being in elementary, middle, or high school (grade range 3–12). Among youth who were out of school, 63% were high school graduates pursuing higher education (Table 2).

**Table 2.** Patient Demographic Characteristics (*N*=139)

Characteristics	
Age at enrollment (years), mean±SD	17.5±3.1
Age (years) at clinic presentation, mean±SD	16.7±3.0
Age distribution (years)	
8–10	5 (3.6)
11–13	12 (8.6)
14–16	36 (25.9)
17–19	53 (38.1)
20–21	33 (23.7)
Assigned sex at birth	
Male	42 (30.2)
Female	97 (69.8)
Gender identity	
Trans masculine spectrum	90 (64.7)
Trans feminine spectrum	40 (28.8)
Nonbinary/gender nonconforming	9 (6.5)
Race	
White	105 (89.7)
Black or African American	4 (3.4)
Asian or Pacific Islander	2 (1.7)
More than one race	6 (5.1)
Ethnicity	
Hispanic or Latino	8 (7.1)
Non-Hispanic or Latino	104 (92.9)
Education level—youth in school	
Elementary	2 (4.6)
Middle	7 (15.9)
High	35 (79.6)
Education level—youth out of school	
Receiving homeschool education	3 (5.9)
Taking time off	1 (2.0)
Formally withdrew (no diploma)	1 (2.0)
High school graduate or equivalent	14 (27.5)
Pursuing higher education	32 (62.8)

Values are mean±SD for continuous variables and *n* (column %) for categorical variables; numbers may not sum to totals due to missing data; column percentages may not sum to 100% due to rounding.

SD, standard deviation.

In terms of sex assigned at birth, 70% of youth were assigned female. Approximately two-thirds of the sample identified on the trans masculine gender identity spectrum (i.e., boy, man, male, trans man, and trans masculine; *n*=90), 29% identified on the trans feminine spectrum (*n*=40), and 7% of youth identified with a nonbinary, gender nonconforming (GNC), or GE identity (*n*=9) (Table 2). Some patients changed the language used to describe gender identity over time (e.g., male vs. trans male). Several patients identified with both binary and nonbinary gender identities at select visits (e.g., female and GNC), or fluctuated between binary and nonbinary gender identities, but gender identity spectrum remained relatively consistent across all visits.

Eighty-eight percent of youth reported that they had socially transitioned, and 89% of youth were reportedly medically transitioning. Of these youth, 30% have or were using GnRH analogs, 76% were receiving GAHT, and 14% have had a gender-confirming surgical procedure (Table 3).

**Table 3.** Patient Clinical Characteristics Related to Gender Identity and Transition

Characteristics	*N* (%)
Socially transitioned^a^	
Yes	121 (87.7)
No	17 (12.3)
Medically transitioning^b^	
Yes	123 (89.1)
No	16 (11.6)
Pubertal blocker use^c^	
Yes	41 (29.5)
No	98 (70.5)
Gender-affirming hormone therapy^c^	
Yes	106 (76.3)
No	33 (23.7)
Gender-affirming surgery	
Yes	20 (14.4)
No	119 (85.6)

Numbers may not sum to totals due to missing data; column percentages may not sum to 100% due to rounding.

^a^ Social transition was defined in terms of gender identity presentation (i.e., pronoun use, use of preferred name, dress) both at home and in public (i.e., school or workplace).

^b^ Medical transition was defined in terms of use of GnRH analogs, gender-affirming hormone therapy and/or surgery.

^c^ Includes both past and current use of pubertal blockers and gender-affirming hormone therapies.

Rates of gender-affirming medical therapy desistence were low. One patient in the registry reverted to a gender identity concordant with their assigned sex at birth after treatment with both GnRH analogs and hormone therapy. Another patient stopped GnRH analogs for a number of years and subsequently resumed treatment. A third patient stopped hormone therapy, but has continued on GnRH analogs while exploring an emerging gender identity. A fourth patient desisted after taking GnRH analogs.

**FIG. 6.**
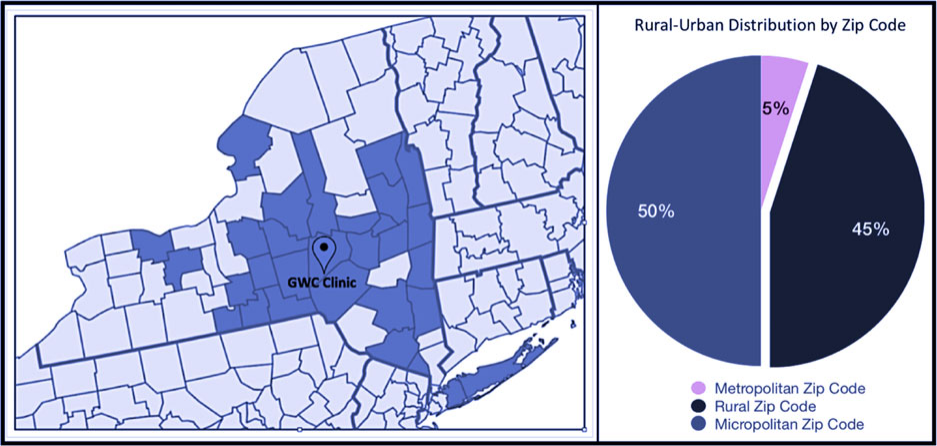
Geographic distribution of GWC patients by county—New York State. This figure shows the catchment area of the GWC registry, which includes 30 counties. Forty-five percent of patients reside in a rural area. Approximately 21% of cities and towns served by the GWC have a population <2,500, 36% have a population <5,000, and 51% have a population <10,000. Fifty percent of patients reside in a micropolitan area, which contains an urban core of at least 10,000 people and a population <50,000; 5% of patients reside in a metropolitan area, defined as an area with a population exceeding 50,000.^30^ GWC, Gender Wellness Center.

Figure 6 shows the geographic distribution of the patient population included in the registry by county. This distribution reflects the need for gender-affirming treatment in many areas of New York state, and the long distances patients must travel to access care. A GWC patient satisfaction survey recently found that more than 60% of patients travel 1–2 h each way for appointments.

## Discussion

This article describes the process of establishing one of the first rural-based patient registries of TG/GE youth. The establishment of the registry is momentous given the dearth of clinical studies investigating the experiences of rural TG/GE people. The GWC catchment area spans a large portion of New York State, drawing patients from both urban and rural areas, hours away from the clinic. The registry has enabled systematic, longitudinal study of this underrepresented population, increasing understanding of the healthcare needs of the youth served by the clinic and filling gaps in the existing evidence based on the treatment of gender dysphoria and incongruence in youth.

### Successes

Our registry recruitment strategy contributed to successful enrollment and low refusal rates. Recruitment took place at GWC appointments with no extra visits required, minimizing the participation burden and facilitating recruitment planning by research staff. Having familiar GWC clinicians initially approach patients helps to communicate that the registry is a GWC clinician-driven research project designed to answer important clinical questions.

A major strength of this registry is the strategic three-step approach that was utilized to create it, specify research questions, and abstract EMR data. The inclusion of qualitative data fields, particularly those describing patient clinical characteristics such as social and medical transition, helps to capture a more comprehensive picture of patient health. Balancing the collection of quantitative and qualitative, narrative-form data was a priority in registry design.

What is perhaps the greatest advantage of the registry for research on TG/GE youth is its suitability to longitudinal studies. The larger field of TG health research is limited by small sample sizes and short follow-up. Our registry has systematically aggregated information about the health of over 139 TG/GE youth, combining both retrospective and prospective data collection.

### Challenges

There are inherent methodological limitations to reliance on medical records as the primary source of data. EMRs are sometimes missing information. Clinician documentation of certain data elements varies in terms of data quality, validity, and comprehensiveness in unstructured fields. Prevalence estimates for abuse, family history of mental illness, and family history of substance use disorder are likely underestimated due to the highly sensitive nature of these topics and lack of disclosure by patients, parents, and guardians at clinic visits.

The process of abstracting comprehensive information from patient EMRs is laborious. The establishment and maintenance of a registry requires personnel, which constitutes one of the major expenses. Additional funds are also required to purchase licenses for survey use. An alternative to using a registry as a tool for longitudinal data collection is the development of queries that can be run on EMRs to abstract data from specific patients. This alternative, however, does not provide the same breadth, depth, or standardized context that a registry offers. Often the results of these queries need to be reviewed and validated using other EMR data or record review. Developing such queries within EMR platforms can also be labor-intensive and complex, and does not offer the flexibility that a stand-alone registry does in terms of altering, adding, and changing data fields for capture.

### Lessons

The creation of a registry of TG/GE youth required careful planning and consideration of research ethics, particularly concerning the potential for identification of registry participants. A number of safeguards are in place not only at the GWC clinic itself but also in the registry recruitment protocol and database design to ensure patient confidentiality. GWC clinicians help to identify and recruit patients for the registry, and their involvement helps to communicate to patients not only that the registry is clinician-driven but also that their data contained in the registry receive the same level of protection as data in their EMR. In addition, all information in the registry is de-identified. Also, REDCap is HIPAA-compliant. Given that ∼45% of registry patients served by the GWC reside in rural areas, and particular consideration has been given to the use of geographical information in publication of registry results. The only geographic information collected in the registry is zip code. The geographic distribution of the GWC patient population is presented at the county level only. We advise that centers considering the establishment of any registry, but in particular a registry of youth for whom disclosure of gender identity could have grave consequences, devote a substantial amount of time and effort to identifying and mitigating potential risks of registry participation. We suggest consulting not only with internal review boards but also with ethics and information technology specialists to ensure that all proper protections are in place.

## Conclusions

As one of the first rural-based registries, this registry has helped to delineate health outcomes related to gender affirming care among a unique patient population of TG/GE youth. We will continue to recruit patients, and over time, we hope to answer all 10 of the original research questions defined by GWC clinicians during the registry building process. We are actively exploring options to expand the registry to other clinics serving TG/GE youth and increase the registry's geographic scope. Our results will be used to describe treatment outcomes that will contribute to evidence-based guidelines.

## Abbreviations Used

CHQ-87: Child Health Questionnaire-87
EMR: electronic medical record
GE: gender expansive
GnRH: Gonadotropin Releasing Hormone
GWC: Gender Wellness Center
HAC: Horizon Ambulatory Care
HIPAA: Health Insurance Portability and Accountability Act
HRQoL: health-related quality of life
ICD: International Statistical Classification of Diseases and Related Health Problems
LAR: legally authorized representatives
REDCap: Research Electronic Data Capture
SF-36: Short Form-36
SOGI: sexual orientation or gender identity
TG: transgender
